# Effect of mental fatigue on the central nervous system: an electroencephalography study

**DOI:** 10.1186/1744-9081-8-48

**Published:** 2012-09-06

**Authors:** Masaaki Tanaka, Yoshihito Shigihara, Akira Ishii, Masami Funakura, Etsuko Kanai, Yasuyoshi Watanabe

**Affiliations:** 1 Department of Physiology, Osaka City University Graduate School of Medicine, 1-4-3 Asahimachi, Abeno-ku, Osaka 545-8585, Japan; 2 Degital & Network Technology Development Center, Panasonic Corporation, 1006 Kadoma, Osaka 571-8501, Japan; 3 RIKEN, Center for Molecular Imaging Science, 6-7-3 Minatojima-minamimachi, Chuo-ku, Hyogo 650-0047, Japan

**Keywords:** Central nervous system, Electroencephalography, Mental fatigue, N-back Test

## Abstract

**Background:**

Fatigue can be classified as mental and physical depending on its cause, and each type of fatigue has a multi-factorial nature. We examined the effect of mental fatigue on the central nervous system using electroencephalography (EEG) in eighteen healthy male volunteers.

**Methods:**

After enrollment, subjects were randomly assigned to two groups in a single-blinded, crossover fashion to perform two types of mental fatigue-inducing experiments. Each experiment consisted of four 30-min fatigue-inducing 0- or 2-back test sessions and two evaluation sessions performed just before and after the fatigue-inducing sessions. During the evaluation session, the participants were assessed using EEG. Eleven electrodes were attached to the head skin, from positions F3, Fz, F4, C3, Cz, C4, P3, Pz, P4, O1, and O2.

**Results:**

In the 2-back test, the beta power density on the Pz electrode and the alpha power densities on the P3 and O2 electrodes were decreased, and the theta power density on the Cz electrode was increased after the fatigue-inducing mental task sessions. In the 0-back test, no electrodes were altered after the fatigue-inducing sessions.

**Conclusions:**

Different types of mental fatigue produced different kinds of alterations of the spontaneous EEG variables. Our findings provide new perspectives on the neural mechanisms underlying mental fatigue.

## Background

Fatigue is a common symptom. In Japan, more than half of the general adult population suffers from fatigue [1]. Fatigue decreases efficiency in the performance of daily activities. In addition, fatigue is one of contributing factors for various medical conditions such as cardiovascular diseases [2], epileptic seizures [3], and Karoshi (death from overwork) [4]. It would thus be of great interest to clarify the mechanisms underlying fatigue and to develop efficient methods for overcoming it. However, the neural mechanisms of fatigue are not well understood.

Fatigue is classified as physical or mental. Physical fatigue is a bodily weakness that can occur because of repetitive muscle activity. In contrast, mental fatigue is observed as a reduced efficiency for mental tasks [5]. Recently, new methods of induction and evaluation of mental fatigue have been proposed [6]. In a mental-fatigue-inducing task session, participants performed 0- or 2-back test trials [7]. The 0-back test was used to represent a lower mental-load task, which could be performed without use of working memory, while the 2-back test was used to represent a higher mental-load task, which could not be performed without using working memory [8]. The advantage of using these tasks is in their ability to cause different types of mental fatigue. Since mental fatigue is a multi-faceted problem [5], it is of great importance to cause mental fatigue using different types of tasks. As a fatigue evaluation mental task session, participants performed cognitive tasks, which are computer-based mental function tasks and the participants were required to use simple and conflict-controlling selective attention. After the 0- or 2-back test sessions, error rates of the evaluation tasks were increased, thus demonstrating a deterioration of the task performance. Task performances were used to assess mental fatigue, and the reliability and validity of the evaluation tasks were satisfactory.

Although a variety of psychophysiological parameters have been used in previous research dealing with fatigue, spontaneous electroencephalography (EEG) has been proposed as the most promising indicator of fatigue [9]. The electrical activity of the brain is classified according to rhythms, which are defined according to frequency bands, including beta, alpha, theta, and delta, and each frequency band is associated with specific internal information processing in the central nervous system [10]. Therefore, alterations of resting-state EEG power induced by mental fatigue may provide valuable clues to identify its neural mechanisms. The aim of our study was thus to clarify the neural underpinnings of mental fatigue using EEG.

## Methods

### Participants

Eighteen healthy male volunteers [30.1 ± 10.8 years of age (mean ± SD)] were enrolled in this study. Current smokers, participants having a history of medical illness, taking chronic medications or supplemental vitamins, or with a body weight less than 40 kg were excluded from the study based on our previous studies [11-15]. The study protocol was approved by the Ethics Committee of Osaka City University, and all the participants provided written, informed consent.

### Experimental design

**Figure 1:**
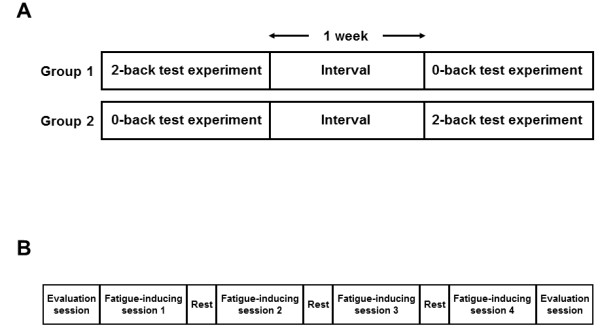
**Experimental design (A) and procedures during experimental sessions (B). **Participants were randomly assigned to two groups in a crossover fashion to perform two types of fatigue-inducing n-back test experiments on separate days. The time interval between each experiment was 1 week. Each experiment consisted of four 30-min fatigue-inducing mental task sessions and two evaluation sessions performed just before and after the four fatigue-inducing sessions.

After enrollment, the participants were randomly assigned to two groups in a single-blinded, crossover fashion to perform two types of fatigue-inducing experiments on separate days (Figure 1A). The time interval between each experiment was approximately 1 week. Each experiment consisted of four 30-min mental-fatigue-inducing task sessions and two evaluation sessions performed just before and after the fatigue-inducing sessions (Figure 1B). During the evaluation session, subjects were evaluated using EEG and electrocardiography (ECG) with their eyes closed for 1 min sitting quietly. Subjects performed cognitive task trials for 9 min, and were then asked to rate their subjective level of fatigue on a Visual analogue scale (VAS) from 0 (minimum) to 100 (maximum) [16]. Saliva samples were collected. This study was conducted in a room at Osaka City University Graduate School of Medicine under quiet, temperature- and humidity-controlled conditions. For 1 day before each session, subjects refrained from intense mental and physical activities, consumed a normal diet and beverages (excluding caffeinated beverages), and maintained normal sleeping hours. They had breakfast just before the session.

### Fatigue-inducing mental task sessions

Participants performed a 0-back or 2-back test for 30 min four times as fatigue-inducing mental task sessions [7]. During this task, one of four letters was presented for 1 s on a display of a personal computer every 3 s. In the 0-back test trial, participants were asked to press the right button with their right middle finger if the target letter (shown beside the personal computer) was presented at the center of the screen. If any other letters appeared, they were to press the left button with their right index finger. In the 2-back test trial, they had to judge whether the target letter presented at the center of the screen was the same as the one that had appeared two presentations before. If it was the same, they were to press the right button with their right middle finger. If it was not the same, they were to press the left button with their right index finger. They were instructed to perform the task trials as quickly and as correctly as possible. The result of each n-back trial, that is, a correct response or error, was continuously presented on the display of the personal computer.

### Cognitive tasks

The cognitive task presentation consisted of traffic lights (placed on a letter corresponding to blue or red in Japanese) and traffic signs for walkers (right or left) and turns (right or left) shown on a personal computer screen. Participants performed Task 1 for 3 min and Task 2 for 6 min. In Task 1, participants were told to press the right button with their right middle finger if the blue traffic light was presented (placed on a letter corresponding to blue in Japanese) regardless of traffic signs for walkers or turns. If the red traffic light was presented, participants were told to press the left button with their right index finger. In Task 2, subjects had to judge whether the target letter presented at the center of a traffic light was blue or red. If the letter meant blue in Japanese**,** regardless of the color of the traffic light or traffic signs for walkers or turns, they were to press the right button with their right middle finger; otherwise, they were to press the left button with their right index finger. The Stroop trial (mismatching the color of the traffic light with the letter) and the non-Stroop trial (matching the color of the traffic light with the letter) occurred equally. In these tasks, each trial was presented 100 ms after pressing either of the buttons. During the task period, blue or red trials and traffic signs for walkers (right or left) and turns (right or left) were given randomly, and the occurrence of each color and type of sign was equal. Subjects were instructed to perform the task trials as quickly and as correctly as possible. The result of each cognitive task trial, that is, a correct response or error, was continuously presented on the display of the personal computer.

### Electroencephalography

EEG was performed using an EEG system (Neurofax μ EEG-9100; Nihon Kohden Corporation, Tokyo, Japan). Eleven electrodes (Ag/AgCl) were attached to the head skin, from positions F3, Fz, F4, C3, Cz, C4, P3, Pz, P4, O1, and O2; and electrooculography (EOG) was also measured to evaluate ocular artifacts. All the electrodes were referenced to linked earlobes. Electrode impedance was maintained below 5 kΩ during the experiment. The EEG was amplified with a 0.3-s time constant and a 120-Hz low-pass filter, and sampled at 500 Hz. Prior to frequency analysis, all EEG data were divided into each epoch, with a duration of 1 s. The recorded data were visually inspected and data segments containing possible residual artifacts were eliminated. EEG larger than +50 μV were rejected as artifact. EOG artifact was also removed by using EOG signals as predictors of the artifact voltages at each EEG electrode. After artifact detection, the data were subjected to a fast Fourier transform, and after averaging, the power was determined in four frequency bands, beta (13–25 Hz), alpha (8–13 Hz), theta (4–8 Hz), and delta (1–4 Hz), for each participant, electrode, and epoch. The average power densities in these frequency bands were log-transformed (ln) for normalization [17].

### Electrocardiography

ECG was recorded using active tracer AC301 (Global Medical Solution Inc., Tokyo, Japan), and the ECG was analyzed using MemCalc for Windows (Global Medical Solution Inc.). Data were analyzed offline after analogue-to-digital conversion at 250 Hz. R-R wave variability was measured as an indicator of autonomic nerve activity. For frequency domain analyses of the R-R wave intervals, low-frequency power (LF) was calculated as the power within the frequency range of 0.04 to 0.15 Hz, high-frequency power (HF) was calculated as that within the frequency range of 0.15 to 0.4 Hz. LF and HF were measured in normalized units. Normalization was performed by dividing the absolute power by the total variance then multiplying by 100. The %HF is vagally mediated [18-20], but the %LF originates from a variety of sympathetic and vagal mechanisms [19,21]. The LF/HF ratio is considered an index of sympathetic nervous system activity [22].

### Saliva sample analyses

We measured saliva cortisol level in order to examine whether the n-back test sessions cause stress response. Saliva samples for the analysis of cortisol were collected in a tube (Salivette; Sarstedt, Rommelsdorf, Germany) and kept on ice until centrifuged at 1700 g for 5 min at 4°C. These supernatants were stored at −80°C until analyzed. The assay for cortisol level was performed by Special Reference Laboratories (SRL; Tokyo, Japan).

### Statistical analyses

The paired *t*-test was used to evaluate the significance of differences between the two conditions. All P values were two-tailed, and values less than 0.05 were considered to be statistically significant. Statistical analyses were performed using the SPSS 17.0 software package (SPSS, Chicago, IL).

## Results

Subjective levels of fatigue, cognitive task performances, ECG parameters and saliva cortisol levels for the fatigue-inducing n-back test sessions are summarized in Table 1. VAS scores of general and mental fatigue were significantly increased after the 0- and 2-back test sessions. As for the cognitive task performances, error rates of Task 2 were significantly increased after the 0- and 2-back test sessions. As for the ECG variables, the LF/HF ratio was increased after the 2-back test sessions although this ratio was not altered after the 0-back test sessions. Saliva cortisol levels were not altered after the 0- or 2-back test sessions.

**Table 1:** Measurements before and after the fatigue-inducing mental task sessions

	**0-back test**		**2-back test**	
	**Before**	**After**	**Before**	**After**
VAS for fatigue				
General fatigue	15.8 ± 11.2	53.2 ± 24.2^a^	14.5 ± 10.4	47.8 ± 23.0^a^
Mental fatigue	15.2 ± 9.9	50.9 ± 27.5^a^	13.2 ± 10.0	47.0 ± 26.0^a^
Cognitive tasks				
Error rate of Task 1	2.4 ± 1.9	3.4 ± 3.3	2.6 ± 2.1	3.7 ± 3.7
Error rate of Task 2	4.4 ± 3.3	6.8 ± 4.9^a^	5.1 ± 4.0	7.1 ± 5.2^a^
ECG				
LF/HF	2.8 ± 5.2	3.7 ± 2.4	1.7 ± 1.0	4.2 ± 3.8^b^
%LF (%)	32.3 ± 16.7	43.2 ± 17.8	34.8 ± 14.9	40.0 ± 23.2
%HF (%)	32.0 ± 25.0	18.3 ± 13.6^b^	26.2 ± 14.1	18.2 ± 17.2
Saliva cortisol (nmol/l)	9.4 ± 4.5	9.4 ± 4.5	8.7 ± 3.3	7.2 ± 3.3

Data are presented as mean ± SD.

VAS, visual analogue scale; LF, low-frequency power; HF high-frequency power.

^a^*P* < 0.01, ^b^*P* < 0.05, significantly different from the corresponding values before the fatigue-inducing mental task sessions (paired *t*-test).

**Figure 2:**
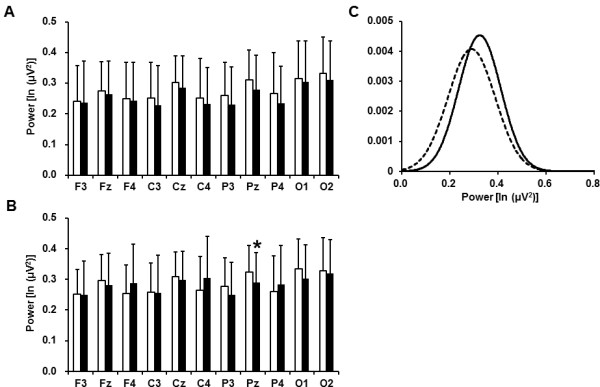
**Electroencephalographic beta power densities before (open columns) and after (closed columns) 2- (A) and 0-back (B) test sessions and Gaussian distributions of the power densities on the Pz electrode before (solid line) and after (dotted line) 2-back test session (C). **Data are presented as mean and SD. ^a^P < 0.05, significantly different from the corresponding values before the fatigue-inducing sessions (paired *t*-test).

The spontaneous EEG beta power densities before and after the fatigue-inducing mental task sessions are shown in Figure 2. In the 2-back test, the beta power density on the Pz electrode was significantly decreased after the fatigue-inducing mental task sessions. In the 0-back test, the beta power densities were not altered on any of the electrodes after the fatigue-inducing task sessions.

**Figure 3:**
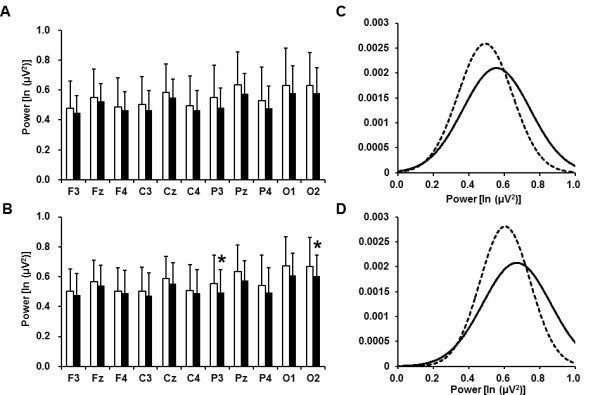
**Electroencephalographic alpha power densities before (open columns) and after (closed columns) 2- (A) and 0-back (B) test sessions and Gaussian distributions of the power densities on the P3 (C) and O2 (D) electrodes before (solid lines) and after (dotted lines) 2-back test session. **Data are presented as mean and SD. ^a^P < 0.05, significantly different from the corresponding values before the fatigue-inducing sessions (paired *t*-test).

The EEG alpha power densities before and after the fatigue-inducing mental task sessions are shown in Figure 3. In the 2-back test, the alpha power densities on the P3 and O2 electrodes were significantly decreased after the fatigue-inducing mental task sessions.

**Figure 4:**
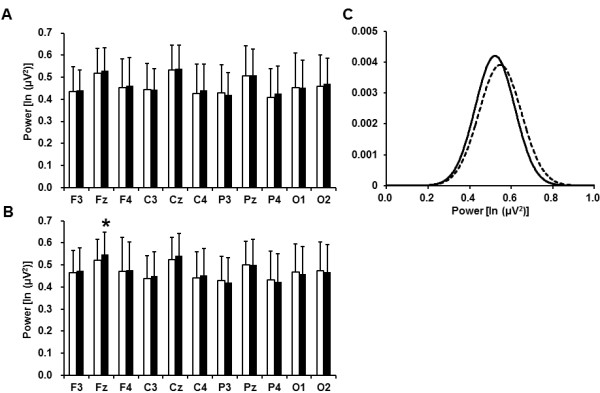
**Electroencephalographic theta power densities before (open columns) and after (closed columns) 2- (A) and 0-back (B) test sessions and Gaussian distributions of the power densities on the Fz electrode before (solid line) and after (dotted line) 2-back test session (C). **Data are presented as mean and SD. ^c^P < 0.05, significantly different from the corresponding values before the fatigue-inducing sessions (paired *t*-test).

The EEG theta power densities before and after the fatigue-inducing mental task sessions are shown in Figure 4. In the 2-back test, the theta power density on the Fz electrode was significantly increased after the fatigue-inducing mental task sessions. In the 0-back test, the theta power densities were not altered on any of the electrodes after the fatigue-inducing task sessions.

The theta/beta and theta/alpha ratios before and after the fatigue-inducing mental task sessions are also evaluated. In the 2-back test, the theta/beta ratios on the Fz (before, 1.89 ± 0.55, after, 2.26 ± 1.11; P = 0.044), Pz (before, 1.62 ± 0.46, after, 1.97 ± 0.95; P = 0.047), and O1 (before, 1.47 ± 0.49, after, 1.75 ± 0.82; P = 0.011) electrodes were significantly increased after the fatigue-inducing mental task sessions, although the theta/alpha ratio did not show any alterations. In the 0-back test, the theta/beta and theta/alpha ratios were not altered on any of the electrodes after the fatigue-inducing task sessions.

**Figure 5:**
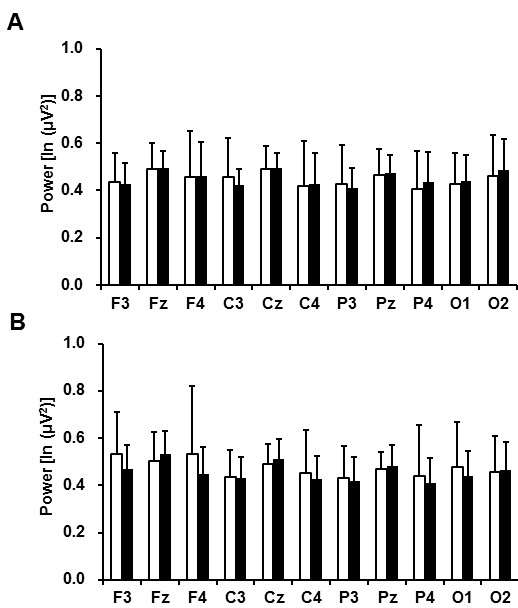
**Electroencephalographic delta power densities before (open columns) and after (closed columns) 2- (A) and 0-back (B) test sessions. **Data are presented as mean and SD.

Finally, the EEG delta power densities before and after the fatigue-inducing mental task sessions are shown in Figure 5. In the 0- and 2-back tests, the delta power densities were not altered on any of the electrodes after the fatigue-inducing task sessions.

## Discussion

We found that in the 2-back test, the beta power density on the Pz electrode and the alpha power densities on the P3 and O2 electrodes were decreased, and the theta power density on the Cz electrode was increased after the fatigue-inducing mental task sessions, while in the 0-back test, no electrodes were altered after the fatigue-inducing sessions.

To confirm that the participants were actually fatigued as a result of performing n-back test trials, they performed cognitive task trials and rated their subjective level of fatigue just before and after the n-back test sessions. After the n-back test sessions, error rates of Tasks 2 were increased (Table 1). These findings are consistent with those of our previous studies [6,23]. In addition, the VAS scores for general and mental fatigue were increased after the sessions (Table 1). These findings demonstrate that the participants were markedly fatigued after the n-back test sessions, and also demonstrate the validity of using the n-back test sessions as fatigue-inducing. No alterations of the saliva cortisol levels demonstrate that the n-back test sessions induced fatigue without or minimum influence of stress or stress response.

The theta power density on the Fz electrode was increased after 2-back task sessions. This finding is consistent with the results of the previous studies, in which fatigue was caused by performing a monotonous simulation driving task [24] or Stroop neuropsychological test [25] for 90 min without any break: In these studies, the theta power density on the frontal EEG electrode site was increased after the mental fatigue-inducing task trials. It has been reported that the theta power density is positively related to sleepiness [26,27]. Thus, alteration of the theta power density in our study may be caused by sleepiness. In fact, the subjective level of sleepiness was increased after the fatigue-inducing mental task sessions (data not shown). Since sleep is one of the most efficient strategies to recover from fatigue, the sleepiness caused by mental fatigue may reflect internal processes designed to meet the demand to recover from mental fatigue. Alternatively, since theta oscillations arising from predominantly fronto-central sources are increased by working memory load [10,28], alteration of the theta power density may be caused just by working memory load caused by performing 30-min 2-back test trials.

In the 2-back test, the beta power density on the Pz electrode and the alpha power densities on the P3 and O2 electrodes were decreased after the fatigue-inducing mental task sessions. Our results of the decreased beta and alpha power densities are consistent with the results of previous studies: The beta power density was decreased by performing a monotonous simulation driving task for 90 min without break [24]; while the alpha power density was decreased by keeping awake and active overnight [29]. While local synchronization in the brain during information processing evolved in the gamma frequency range, synchronization between neighboring cortices during multi-modal information processing evolved in the beta frequency range, and long range interactions during high-level information processing such as visuospatial attention evolved in the alpha frequency range [30]. Since multi-modal and high-level information processing are associated with the beta and alpha power densities, respectively, decreased beta and alpha power densities under conditions of mental fatigue indicate deterioration of multi-modal and high-level information processing in the central nervous system. Our results for the cognitive tasks (Table 1) support this speculation.

Different types of mental fatigue produced different styles of the alterations of the EEG variables: in the 2-back test, the beta power density on the Pz electrode and the alpha power densities on the P3 and O2 electrodes were decreased, and the theta power density on the Fz electrode was increased. In the 0-back test,no electrodes were altered after the fatigue-inducing sessions. The 0-back test was used to represent a lower mental-load task, which could be performed without working memory use, while the 2-back test was used to represent a higher mental-load task, which could not be performed without working memory use [7]. Most of the locations that showed changes of beta and alpha power densities are located close to the visual areas (P3, Pz, and O2) in the 2-back test. Higher mental load to perform 2-back test may need more visual memory and implies more visual work in different visual areas to develop the fatigue-related alterations of EEG power densities in the posterior areas related to visual processing. Higher mental load thus may trigger processes designed to bring about deterioration of multi-modal and high-level information processing, while lower mental load may induce fewer alterations.

In addition to EEG, different types of mental fatigue produced different styles of the alterations of the ECG variables. The LF/HF ratio was increased after the 2-back test sessions although this ratio was not altered after the 0-back test sessions. Since increased LF/HF ratio during mental fatigue-inducing task session was reported to be associated with the mental effort or motivation [23], the different results of the LF/HF ratio between the 0- and 2-back test sessions might result from the difference of the mental effort or motivation between the sessions. The brain network, including the prefrontal cortex (PFC) and anterior cingulate cortex (ACC), has been shown to play an important role in the regulation of autonomic nervous activities [31]. Abnormalities in these brain regions have been shown to be associated with fatigue [32,33]. Because impaired selective attention assessed by increased error rates in cognitive task trials were observed after the fatigue-inducing mental task sessions, and the selective attention process activates the PFC and ACC [34-37], the higher mental load required for the 2-back test sessions might introduce temporary dysfunctions in the PFC and ACC to cause decreased parasympathetic and increased sympathetic activities, while lower mental load necessary for performing the 0-back test sessions might induce fewer alterations of the ECG variables.

### Limitations

While the results of the present study are suggestive of causal relationships between information load and mental fatigue, only a limited number of participants were tested. The ratio of fatigue was similar between men and women in the society [38]. However, it is not clear whether the findings in our study can be considered to be the same in women. To generalize our results, studies involving a larger number of participants will be needed.

## Conclusions

We identified mental fatigue-related changes in spontaneous EEG variables. In the 2-back test, the beta power density on the Pz electrode and the alpha power densities on the P3 and O2 electrodes were decreased, and the theta power density on the Cz electrode was increased after the fatigue-inducing mental task sessions, while in the 0-back test, no electrodes were altered after the fatigue-inducing sessions. Our findings provide new perspectives on the neural mechanisms underlying mental fatigue.

## Abbreviations

ACC: Anterior cingulate cortex; ECG: Electrocardiography; EEG: Electroencephalography; HF: High-frequency power LF, low-frequency power; PFC: Prefrontal cortex; VAS: Visual analogue scale.

## Competing interests

The authors declare that they have no competing interests.

## Authors’ contributions

MT took part in planning and designing the experiment, collected the data, performed the data analyses and drafted the manuscript. YS, AI, MF, and EK took part in planning and designing the experiment, collected the data, and performed the data analyses. YW took part in the planning and designing the experiment and helped drafting the manuscript. All authors read and approved the final manuscript.
